# Amino Acid Substitutions Associated with Avian H5N6 Influenza A Virus Adaptation to Mice

**DOI:** 10.3389/fmicb.2017.01763

**Published:** 2017-09-15

**Authors:** Chunmao Zhang, Zongzheng Zhao, Zhendong Guo, Jiajie Zhang, Jiaming Li, Yifei Yang, Shaoxia Lu, Zhongyi Wang, Min Zhi, Yingying Fu, Xiaoyu Yang, Lina Liu, Yi Zhang, Yuping Hua, Linna Liu, Hongliang Chai, Jun Qian

**Affiliations:** ^1^Military Veterinary Research Institute, Academy of Military Medical Sciences Changchun, China; ^2^College of Wildlife Resources, Northeast Forestry University Harbin, China; ^3^Institute of Chinese Materia Medica, China Academy of Chinese Medical Science Beijing, China

**Keywords:** avian H5N6 influenza A virus, amino acid substitutions, mammalian adaptation, pathogenicity, transmissibility

## Abstract

At least 15 cases of human beings infected with H5N6 have been reported since 2014, of which at least nine were fatal. The highly pathogenic avian H5N6 influenza virus may pose a serious threat to both public health and the poultry industry. However, the molecular features promoting the adaptation of avian H5N6 influenza viruses to mammalian hosts is not well understood. Here, we sequentially passaged an avian H5N6 influenza A virus (A/Northern Shoveler/Ningxia/488-53/2015) 10 times in mice to identify the adaptive amino acid substitutions that confer enhanced virulence to H5N6 in mammals. The 1st and 10th passages of the mouse-adapted H5N6 viruses were named P1 and P10, respectively. P1 and P10 displayed higher pathogenicity in mice than their parent strain. P10 showed significantly higher replication capability *in vivo* and could be detected in the brains of mice, whereas P1 displayed higher replication efficiency in their lungs but was not detectable in the brain. Similar to its parent strain, P10 remained no transmissible between guinea pigs. Using genome sequencing and alignment, multiple amino acid substitutions, including PB2 E627K, PB2 T23I, PA T97I, and HA R239H, were found in the adaptation of H5N6 to mice. In summary, we identified amino acid changes that are associated with H5N6 adaptation to mice.

## Introduction

Due to the antigenic differences of HA and NA, influenza A virus is classified into 18 HA subtypes and 11 NA subtypes. Except for H17N10 and H18N11 isolated from bats (Tong et al., 2013; Zhu et al., 2013), all influenza virus subtypes have been identified in wild birds, which are the natural influenza virus reservoirs (Yoon et al., 2014). Avian influenza viruses replicate efficiently in wild birds, but replicate poorly in other mammals (Yoon et al., 2014). However, a growing body of evidence suggests that avian influenza viruses can infect other mammals, such as cats, dogs, rhesus macaques, and plateau pikas (Shinya et al., 2012; Cheng et al., 2014b; Yu et al., 2014a, 2015). Sporadical H5N1, H7N9, and H10N8 infections in humans have been reported in China, indicating that other avian influenza virus subtypes pose potential threats to humans through the natural evolution (Claas et al., 1998; Gao et al., 2013; Zhang W. et al., 2014).

Avian H5N6 influenza virus was first isolated from mallards in North America in 1975 (Garcia et al., 1997). In China, H5N6 viruses first emerged in 2013 and have widely circulated in wild waterfowl and poultry during recent years (Qi et al., 2014; Bi et al., 2015; Wu et al., 2015; Yu et al., 2015). Unlike H5N2 and H5N8 viruses that are distributed worldwide, H5N6 viruses are largely confined to East and Southeast Asia, including China, Vietnam, Laos, and South Korea (Su et al., 2015; Wong et al., 2015; Kwon et al., 2017). The first identified human case infected with H5N6 was first identified as a human infectious agent in a 5-year-old girl admitted to a sentinel hospital of the Chinese influenza surveillance system in Changsha in 2014 (Zhang et al., 2016), while the first fatal human H5N6 infection occurred in Sichuan Province in 2014 (Pan et al., 2016). The newly identified H5N6 virus isolated in Yunnan Province was a tri-reassortment of H5N1 in ducks, H6N6 in chickens, and H9N2 in poultry (Feng et al., 2016; Xu et al., 2016), of which the six internal genes were all from H9N2 viruses (Feng et al., 2016; Xu et al., 2016). Similarly, avian H7N9 influenza viruses isolated from humans in China in 2013 carried six internal genes from H9N2 viruses (Zhang Y. et al., 2014). More importantly, 10 new cases of H5N6 infecting humans occurred in 2016 in China, indicating a sharp increase in the frequency of human H5N6 infection and raising public health concerns amongst officials. A previous study demonstrated that two H5N6 viruses isolated from ducks and geese could be transmitted among guinea pigs by direct contact (Sun et al., 2016). Taken together, the human cases of H5N6 infection, the transmissibility of H5N6 to mammals and the lack of pre-existing immunity to H5N6 in humans suggest that H5N6 viruses might pose a potential pandemic threat to humans through further mammalian adaptation. Hence, the molecular features involved in the adaptation of H5N6 viruses to mammals must be further elucidated.

Mice are the most widely used animal model for studying the pathogenesis and mammalian adaptation of avian influenza A viruses (Li et al., 2005), for which two practical reason exist. First, mice are convenient models in terms of size, cost, and husbandry requirements, allowing large mouse study sizes and easily obtained statistically robust data (Bouvier and Lowen, 2010). Moreover, the ability to genetically manipulate mice offers a system in which the host response to infection can be studied in detail. However, there are numerous drawbacks in using mouse models, such as the significant susceptibility differences and pathophysiological differences in influenza infection between mice and humans, which could make the model unsuitable for addressing certain virological questions and render data obtained in mice difficult to translate to humans (Bouvier and Lowen, 2010). Serial passage in mice has become a common method to determine the amino acid changes during the adaptation of influenza viruses to mammal hosts (Gabriel et al., 2013; Yu et al., 2014b; Chen et al., 2015; Wu et al., 2016). By studying these amino acid changes using reverse genetics, we aimed to elucidate the molecular features that contribute to the adaptation of influenza A viruses in mammals, conferred enhanced pathogenicity and efficient transmissibility of influenza A viruses in mammals.

In 2015, we isolated a novel H5N6 virus, named A/Northern Shoveler/Ningxia/488-53/2015, from wild birds fences in Ningxia, China. To identify the amino acid changes related to the adaptation of this H5N6 virus to mammal hosts, we sequentially passaged the virus 10 times in mice and identified the amino acid substitutions associated with the adaptation of H5N6 to mice. We also evaluated the pathogenicity and transmissibility of mouse-adapted H5N6 viruses.

## Materials and Methods

### Ethics Statement

All animal studies were conducted in strict accordance with the Guide for the Care and Use of Laboratory Animals of the Military Veterinary Research Institute. All animal experiments were approved by the Animal Care and Use Committee of the Military Veterinary Research Institute.

### Virus

The avian H5N6 influenza virus (A/Northern Shoveler/Ningxia/488-53/2015) was isolated from wild birds fences in Ningxia, China and stored in our laboratory. All viruses were grown in specific-pathogen-free (SPF) embryonated eggs and stored at -80°C. Viruses were titrated in 9-day-old embryonated eggs to determine the median egg infectious dose (EID_50_) using the Reed–Muench method. All experiments involving viruses were performed in Animal Biosafety Level-3 (ABSL-3) facilities.

### Animals

Six-week-old female BALB/c mice weighing 15–16 g and female Hartley strain guinea pigs weighing 300–350 g were purchased from the Vital River Laboratory. All animals used in this study were serologically negative for influenza virus, and were housed in the ABSL-3 facilities.

### Adaptation of Avian H5N6 Influenza Virus in Mice

To identify the amino acid substitutions associated with the adaptation of influenza virus in mammals, the H5N6 influenza virus isolated from wild birds was serially passaged in 6-week-old female BALB/c mice. Briefly, three ether-anesthetized mice were inoculated intranasally (i.n.) with 50 μL of 10^6^ EID_50_/mL H5N6 influenza A virus. The infected mice were sacrificed 72 h after inoculation, and their lung tissues were harvested in 1 mL of PBS, disrupted and centrifuged to collect the homogenized lungs tissue supernatants. Three naïve mice were then inoculated i.n. with 50 μL of homogenized lung tissue supernatants harvested from the previously inoculated mice, and 10 successive passages were performed. The mouse-adapted H5N6 influenza viruses were isolated from the homogenized lung tissue supernatants of the serially passaged mice using 9-day-old SPF embryonated eggs.

### Sequence Analysis

Viral RNAs were extracted from the homogenized lung tissue supernatants using an RNeasy Mini-kit (Qiagen, Germany) according to manufacturer’s protocol. Viral RNAs were reverse transcribed into cDNAs using influenza virus-specific U12 primers and an RT reagent kit (Takara, Japan) and viral genes were amplified using a PCR kit (Takara, Japan) according to the manufacturer’s protocol. The PCR reaction conditions were as follows: pre-denaturation at 94°C for 5 min, then followed by 35 cycles of 94°C for 40 s, 55°C for 40 s, 72°C for 2 min, prior to a final extension step at 72°C for 10 min. The primers used for PCR are listed in Supplementary Table S1. The PCR products were sequenced by the Comate Bioscience Corporation (Changchun, China). The influenza virus genomic sequences were translated into proteins, and the protein sequences resulting from the 10 passages were aligned with those of H5N6 isolated from wild birds using DNAman software (Lynnon Biosoft, United States). Finally, amino acid substitutions in the different H5N6 viral passages were identified, and the eight wild type (WT) H5N6 gene segments were deposited into the GenBank database under the accession numbers MF399665–MF399672.

### Mouse Challenged Pathogenicity Studies

To evaluate the pathogenicity of the mouse-adapted H5N6 influenza viruses, groups of six 6-week-old female BALB/c mice were anesthetized with ether and inoculated i.n. with 50 μL of two mouse-adapted viruses passage 1 (P1) and passage 10 (P10) and WT H5N6 virus at 10^6^ EID_50_/mL. PBS by itself was used as the control. Body weight changes and mouse survival rate were monitored daily for 14 days, and mice that lost more than 25% of their body weight were euthanized.

To detect the tissue distribution of mouse-adapted influenza virus variants *in vivo*, groups of 12 six-week-old female BALB/c mice were inoculated i.n. with 50 μL of the P1, P10, and WT H5N6 virus at 10^6^ EID_50_/mL, while three naïve mice were inoculated with PBS as control. On days 3 and 5 post-infection (dpi), three mice were sacrificed and tissue samples, including lung, heart, kidney, spleen, liver, and brain tissues, were harvested in 1 mL of PBS. These tissue samples were then homogenized and centrifuged, viral titers in the homogenized tissue supernatants were determined by the Reed–Muench method using 9-day-old embryonated eggs.

To assess pathological lesions from mice infected with the mouse-adapted influenza virus variants, their lungs were harvested at 3 dpi and fixed with 4% formalin. The sectioned lungs were stained with hematoxylin and eosin and then subjected to pathological examination with light microscopy.

### Guinea Challenged Transmission Studies

To measure the contact transmissibility of a mouse-adapted influenza virus variant, three guinea pigs were inoculated i.n. with 200 μL of P10 virus (100 μL in each nostril) at 10^6^ EID_50_/mL and then housed in a cage inside an isolator. Three naïve guinea pigs were co-housed with the three infected guinea pigs 24 h post-inoculation, and nasal washes were collected from each guinea pig every other day, beginning at 2 dpi. Viruses in the nasal washes were titrated in 9-day-old embryonated eggs and the experiment conditions for these studies were set at 19–23°C with 30–50% relative humidity.

To evaluate the aerosol transmissibility of a mouse-adapted influenza virus variant, three guinea pigs were inoculated i.n. with 200 μL of P10 virus in PBS (100 μL in each nostril) at 10^6^ EID_50_/mL. Each inoculated guinea pig was housed in a separate cage placed in an independent isolator. The next day, three naïve guinea pigs were paired with the three inoculated guinea pigs. Each naïve guinea pig was housed in a new cage adjacent to the cage housing its respective infected partner, and the distance between the two adjacent cages was 5 cm apart. Nasal washes were collected from each guinea pig every 2 days, beginning at 2 dpi. Viruses were titrated in embryonated eggs.

### Statistical Analysis

Quantitative data were analyzed with GraphPad Prism 5.0 software using the one-way or two-way ANOVA method, and statistical comparisons between two groups were tested using the Student–Newman–Keuls (SNK) method. Survival analysis data were analyzed with GraphPad Prism 5.0 software using log-rank tests. *P*-values less than 0.05 indicated significant differences.

## Results

### Mouse-Adapted H5N6 Viruses Showed Enhanced Pathogenicity in Mice

To study the adaptation of H5N6 virus isolated from wild birds to mammals, we serially passaged WT H5N6 influenza A virus in mice. Three female BALB/c mice (passage 1) were first inoculated i.n. with 50 μL of 10^6^ EID_50_/mL WT H5N6. Three days later, the infected mice were sacrificed, and their homogenized lung tissue supernatants were subsequently used to inoculate i.n. the next three mice (passage 2). These procedures were repeated until passage 10, and 10 passages of mouse-adapted H5N6 viruses were isolated from the homogenized lung supernatants.

**FIGURE 1:**
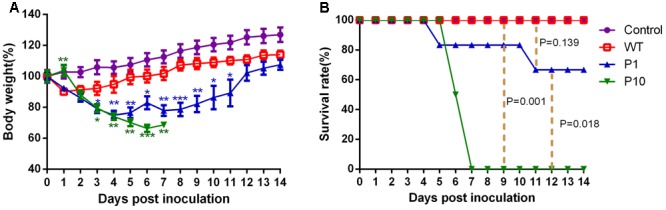
Body weight changes and survival rates of mice inoculated with mouse-adapted H5N6 viruses. Groups of six 6-week-old BALB/c female mice were inoculated i.n. with 50 μL of P10, P1, and WT H5N6 viruses 10^6^ EID_50_/mL of or PBS. Body weight changes and survival rates were daily monitored for 14 days. The mouse body weights were represented as the average weight percentage on the day of inoculation (day 0). The average body weights of each group were shown and error bars represented the standard deviation (SD). **(A)** Percent change in body weight from day 0. Body weight data was analyzed using two-way ANOVA method and the comparisons between two groups were made using SNK method. ^∗^*P* < 0.05; ^∗∗^*P* < 0.01. The blue asterisks represent the significant difference between P1 and WT, while the green asterisks represent the significant difference between P10 and WT. **(B)** Survival rates. Survival data comparisons between each two groups (P10 vs WT, P1 vs WT, and P10 vs P1) were analyzed with GraphPad Prism 5.0 software using the log-rank test method, and the *P*-values are indicated in the graph.

We compared the pathogenicity of WT H5N6 with mouse-adapted P1 and P10 viruses. Groups of six 6-week-old female BALB/c mice were infected with 50 μL of P10, P1, or WT viruses at 10^6^ EID_50_/mL. Body weight loss and mortality were successively monitored for 2 weeks, and the PBS-inoculated group served as the control. All mice infected with WT virus survived, and their average body weight decreased approximately 10% at 1 dpi before gradually recovering to normal (**Figure 1**). In contrast, all mice inoculated with P10 virus rapidly lost weight and succumbed to death by 7 dpi, while mice infected with P1 virus showed moderate pathogenicity (**Figure 1**). The body weights of mice infected with P1 virus were rapidly reduced approximately 25% during the first 4 dpi before finally recovering to the normal state (**Figure 1A**). Two of six mice infected with P1 virus died (**Figure 1B**).

**FIGURE 2:**
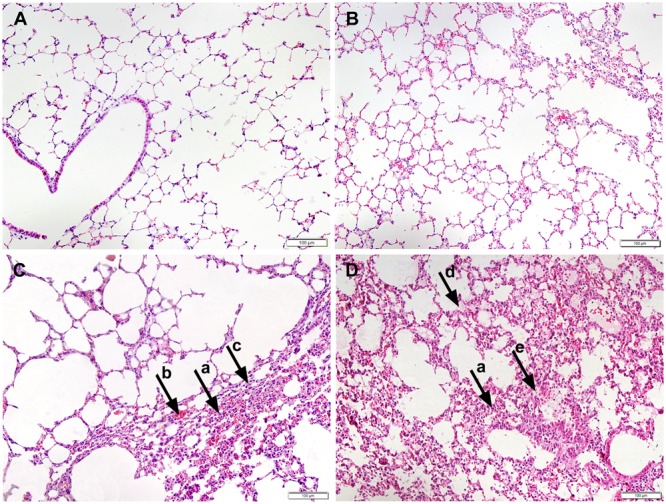
Histopathology of the lungs of mice inoculated with PBS **(A)**, WT H5N6 **(B)**, P1 **(C)**, or P10 **(D)**. The infected mouse lungs were fixed with formalin, embedded in paraffin and stained with hematoxylin and eosin. Images were obtained at 20× magnification; arrow a, neutrophil infiltration; arrow b, alveolar walls capillary hyperemia; arrow c, alveolar wall thickening; arrow d, acidophilic protein-like exudation; arrow e, epithelial cell shedding.

Additionally, the WT-infected mice showed no obvious pathological changes, as determined by histopathological analysis (**Figure 2B**). A slight infiltration of inflammatory cells and alveolar wall thickening was observed in P1-infected mice (**Figure 2C**) while acidophilic protein-like exudation, epithelial cell shedding and moderate pneumonia with neutrophilic granulocyte infiltration were observed in P10-infected mice (**Figure 2D**). No obvious histopathological changes were observed in the negative control (mock infected) mice (**Figure 2A**). Histological analysis demonstrated that the mouse-adapted viruses exhibited more severe histopathological changes than the WT-infected mice.

In summary, the mouse-adapted H5N6 viruses, especially those of P10, showed enhanced pathogenicity in mice, suggesting an adaptation of H5N6 from avians to mammals.

### Mouse-Adapted H5N6 Viruses Replicated to Higher Titers in Lungs and Could be Detected in the Brains of Mice

**FIGURE 3:**
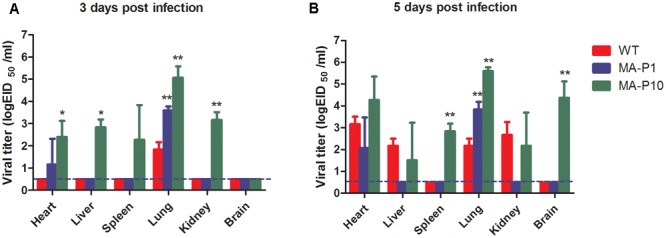
Tissue distribution of mouse-adapted H5N6 viruses. Groups of six 6-week-old female BALB/c mice were infected with 50 μL of the P10, P1, and WT H5N6 viruses at 10^6^ EID_50_/mL. At 3 and 5 dpi, three mice in each group were sacrificed, and different tissue samples, including lung, heart, liver, kidney, spleen, and brain tissues, were homogenized in 1 mL of PBS. Viral titers in the homogenized tissue supernatants were determined by the Reed–Muench method. The results are presented as the mean ± SD, and all data was analyzed using one-way ANOVA method and the comparisons between two groups were made using SNK method; ^∗^*P* < 0.05, and ^∗∗^*P* < 0.01 (P10 vs WT and P1 vs WT). The dashed lines indicate the lower limit of detection. **(A)** Tissue distribution of mouse-adapted H5N6 viruses at 3 dpi. **(B)** Tissue distribution of mouse-adapted H5N6 viruses at 5 dpi.

To determine whether the replication capacities of mouse-adapted H5N6 viruses were significantly enhanced, we compared the replication capabilities and system viral spreads of two mouse-adapted H5N6 viruses with WT virus. Groups of six 6-week-old female BALB/c mice were infected with P10, P1, and WT viruses. At 3 and 5 dpi, three mice were sacrificed and their lungs, hearts, livers, kidneys, spleens, and brains were harvested. Viral titers in the collected tissues were determined by the Reed–Muench method. WT virus could replicate in mouse lungs, with titers ranging from 1.5 to 2.5 log_10_ EID_50_ (**Figure 3**). In contrast, P10 replicated very well in mouse lungs, with titers approximately 5.1 and 5.5 log_10_ EID_50_ at 3 and 5 dpi (**Figure 3**), which was approximately 1000-fold higher than that of WT virus. P1 replicated in the lungs to a titer of 3.6 and 3.8 log_10_ EID_50_ at 3 and 5 dpi (**Figure 3**), which was much higher than that of WT virus. Therefore, the mouse-adapted H5N6 viruses, especially P10, displayed much higher replication capability in mouse lungs. Systemically, in addition to being detected in the lungs of mice, WT virus was detected in their hearts, livers, and kidneys at only 5 dpi, while it was undetectable in their spleens and brains (**Figure 3B**). In sharp contrast, except for the brain at 3 dpi, P10 was found in all tested tissues (**Figure 3A**). Specifically, P10 was detected in the brains of mice at 5 dpi, with a higher titer of 4.4 log_10_ EID_50_ being observed (**Figure 3B**), whereas no virus was detected in the brains of mice infected with P1 in the 7 days of animal experiments. Thus, the mouse-adapted H5N6 viruses, especially the P10 virus, displayed higher replication abilities than their parental virus strain in mice.

### Mouse-Adapted H5N6 Virus Was No Transmissible between Guinea Pigs by Contact or Aerosol

**FIGURE 4:**
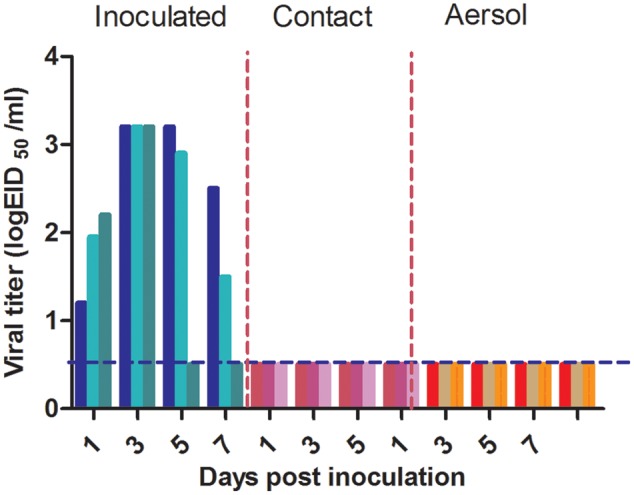
Evaluation of the transmissibility of P10 in guinea pigs. For contact transmission, three guinea pigs were inoculated i.n. with 200 μL of 10^6^ EID_50_/mL P10 virus. Three naïve guinea pigs were co-housed with the three infected guinea pigs 24 h post-inoculation. For aerosol transmission, three guinea pigs were inoculated i.n. with 200 μL of 10^6^ EID_50_/mL P10. The next day, three naïve guinea pigs were paired with the three inoculated guinea pigs. Each naïve guinea pig was housed in a new cage 5 cm from its respective infected partner. Nasal washes were collected from all guinea pigs every 2 days, beginning at 1 day post-exposure. Viruses in the nasal washes were titrated in embryonated eggs. The blue dashed lines indicate the lower limit of detection.

We evaluated the transmissibility of P10 in guinea pigs by contact. Three guinea pigs were inoculated i.n. with 200 μL of 10^6^ EID_50_/mL P10. The next day, three naïve guinea pigs were co-housed with the three infected animals. To assess the virus transmission in guinea pigs, nasal washes from all guinea pigs were collected at 2-day intervals, and the viruses were titrated in 9-day-old embryonated eggs. Viruses were detected in all nasal washes from the inoculated guinea pigs from 1 to 5 days post-contact, but no virus was detected in the three naïve guinea pigs of the contact group (**Figure 4**).

We also measured the aerosol transmissibility of P10 in guinea pigs. Three guinea pigs were inoculated i.n. with 200 μL of 10^6^ EID_50_/mL P10 virus. Twenty-four hours later, three naïve guinea pigs were paired with the three inoculated guinea pigs and each naïve guinea pig was housed in a new cage adjacent to the P10-infected guinea pigs at a distance of 5 cm. Viral titers in the nasal washes collected from all guinea pigs at different time points post-exposure were determined by Reed–Muench method. No virus was found in nasal washes of the naïve guinea pigs of the aerosol group (**Figure 4**). Therefore, similar to its parental strain WT H5N6 strain, P10 did not acquire the ability to be transmitted between guinea pigs by contact or aerosol.

### Amino Acid Substitutions in Mouse-Adapted H5N6 Viruses

To identify the amino acid substitutions associated with the enhanced pathogenicity and replication, the genomes of viruses from all 10 passages were amplified by PCR and sequenced. Eight amino acid substitutions were identified, two in the HA subunit (I67V, R239H), two in the polymerase basic protein 2 (PB2) subunit (T23I, E627K), two in the polymerase acidic (PA) subunit (T97I, S388C) and two in the nucleoprotein (NP) subunit (H52Y, A430T) (**Table 1**). Most (five of eight) amino acid changes, including HA I67V, PA S388C, PB2 E627K, NP H52Y, and NP A430T, first occurred in the P1 virus, indicating that the WT H5N6 virus rapidly adapted in the presence of selective pressure in mice. PB2 T23I was identified in P2, HA R239H was found in P6 and T97I was first observed in P7.

**Table 1:** Amino acid substitutions in the mouse-adapted H5N6 influenza virus.

Segment	Position	WT	P1	P2	P3	P4	P5	P6	P7	P8	P9	P10
HA	**67**	I	**V**	**V**	**V**	**V**	**V**	**V**	**V**	**V**	**V**	**V**
	**239**	R	R	R	R	R	R	**H**	**H**	H	**H**	**H**
NP	**52**	H	**Y**	**Y**	**Y**	**Y**	**Y**	**Y**	**Y**	**Y**	**Y**	**Y**
	**430**	A	**T**	**T**	**T**	**T**	**T**	**T**	**T**	**T**	**T**	**T**
PA	**97**	T	T	T	T	T	T	T	**I**	**I**	**I**	**I**
	**388**	S	**C**	**C**	**C**	**C**	**C**	**C**	**C**	**C**	**C**	**C**
PB2	**23**	T	T	**I**	**I**	**I**	**I**	**I**	**I**	**I**	**I**	**I**
	**627**	E	**K**	**K**	**K**	**K**	**K**	**K**	**K**	**K**	**K**	**K**

*^∗^Viruses isolated from all 10 passages were sequenced. The nucleotide sequences were translated into proteins and aligned to identify amino acid changes. All identified amino acid substitutions are presented here. The amino acids identified after substitutions are displayed in bold.*

The PB2 E627K substitution found in P1 has been identified in many other mouse-adapted influenza viruses, including H5N1, H7N9, and H9N2 (Gabriel et al., 2013; Mok et al., 2014; Sang et al., 2015a). This amino acid substitution was also found in another mouse-adapted H5N6 influenza virus of duck origin, whereas other mutations including PA A343T, NA R143K, and NA G147E described in Peng’s work were not found in our study (Peng et al., 2016). The PB2 E627K substitution is one of the most common amino acid substitutions in different influenza viruses subtypes adapted in mice and occurs at the early stage of influenza A virus mouse adaptation. In the present work, the PB2 E627K mutation played important roles in the early adaptation of the H5N6 virus to mice. PB2 E627K can aid the adaptation of influenza A virus to mammals in two ways. First it can directly alter the temperature-dependent enzyme kinetics of the influenza A virus polymerase complex and improve polymerase efficiency at 33°C (Aggarwal et al., 2011), which was proven for H7N9 (Zhang H. et al., 2014). Additionally, the PB2 E627K mutation could increase the strength of polymerase nucleocapsid binding and modulate nucleocapsid inhibition via the pathogen sensor RIG-I (Weber et al., 2015). These two mechanisms together improve the replication efficiency and virulence of influenza A viruses. Thus, PB2 E627K enhanced the replication capacity and pathogenicity of P1 compared with WT H5N6 (**Figures 1**–**3**). The other four mutations occurred in P1 was first identified in our work and their functions have yet to be explored. Thus the adaptation of different influenza A virus isolates to mice varied greatly.

The PA T97I substitution observed in P7 has also been identified in many other mouse-adapted influenza A viruses, such as H5N2, H6N1, H7N9, and H10N7 (Song et al., 2009; Cheng et al., 2014a; Wu et al., 2016; Zhao et al., 2016). This mutation is a very common substitution in influenza A viruses adapted in mice and occurs during the late influenza virus adaptation stage. Thus PB2 T97I plays important roles in the late adaptation of H5N6 to mice. Alone or in combination with PB2 E627K, PA T97I was shown to enhance the polymerase activity, replication efficiency, and pathogenicity of influenza A virus to aid the adaptation of virus to mice, and this was also true for H5N2 and H7N9 (Cheng et al., 2014a; Zhao et al., 2016). In this study, PA T97I and PB2 E627K together aided the adaptation of WT H5N6 virus to mice and enhanced the pathogenicity of P10. Indeed, the replication and pathogenicity of P10 was higher than that of P1 and WT H5N6. We next determined the replication curves of the P10 and WT H5N6 viruses in MDCK cells. As expected, the replication ability of P10 in MDCK cells was much higher than that of WT H5N6 viruses. Experimental results indirectly proved that the polymerase activities of P10 were much higher than that of WT H5N6 (Supplementary Figure S1).

The PB2 subunit of influenza virus polymerase bound to mitochondrial antiviral signaling protein (MAVS) through its N-terminal domain, specifically to its first 37 amino acids, to inhibit IFN-β expression (Carr et al., 2006; Iwai et al., 2010). The PB2 T23I substitution, which was first identified in P2 in our work, is located in the N-terminal domain of PB2, and we speculated that it might also inhibit IFN-β expression and the immune escape of IFN-β antiviral effects.

R239H (227 in H3 numbering) found in P6 was located in the 220 loop of influenza virus receptor binding sites. A previous study demonstrated that the HA Q227P substitution increased the ability of guinea pig-adapted H9N2 to bind an avian α2,3 receptor and did not affect its binding ability to a human α2,6 receptor, and demonstrated the critical effects of this mutation for influenza virus transmission in guinea pigs by direct contact (Sang et al., 2015b). The side chain groups of proline (P) and histidine (H) are piperidine and imidazole, respectively, both of which are non-polar heterocyclic ring. We speculated that HA R227H could also improve the ability of P10 to bind avian α2,3 receptors and may confer transmissibility to P10. However, P10 remained no transmissible by contact or aerosol in guinea pigs. To determine the reason underlying the lack of P10 transmissibility in guinea pigs, the receptor binding p3s of P10 and WT H5N6 were determined using the chicken red blood preference method. Like its parental WT H5N6 strain, P10 bound only avian α2,3 receptors, but not human α2,6 receptors (Supplementary Figure S2), and binding to human α2,6 receptors is a prerequisite for the efficient transmission of influenza virus in mammals. Hence, the inability of P10 to be transmitted in guinea pigs can be easily attributed to the fact that P10 binds only avian α2,3 receptors.

## Discussion

Considering the potential pandemic threat of avian H5N6 viruses to public health, more attention should be paid to the mammalian adaptive processes of H5N6 viruses. In the present study, we first reported the adaptation of an avian H5N6 influenza virus isolated from wild waterfowl in mice. Multiple amino acid substitutions associated with the adaptation of H5N6 influenza virus to mice were identified, and they conferred enhanced pathogenicity and higher replication capability to H5N6 in mammals.

Most amino acid changes first occurred in P1, the early WT H5N6 passage step in mice indicating that H5N6 isolated from wild birds adapted rapidly in mammals. Wild birds are the natural reservoirs of influenza A viruses and many H5N6 viruses have also been isolated from wild birds (Yoon et al., 2014; Sun et al., 2016). With the expansion of human activity, including tourism and agriculture, an increasing number of people have invaded wild birds habitats, and the chance of humans being infected by influenza A viruses could be significantly increased through direct or indirect contacts with wild birds carrying influenza viruses. Some wild birds originated H5N6 viruses have acquired limited pathogenicity and transmissibility in mammals (Sun et al., 2016). Once viruses enter humans, they quickly adapt and evolve, potentially enhancing the pathogenicity and transmissibility in humans, leading to endemic or pandemic influenza. Thus the continued surveillance of amino acid substitutions in H5N6 viruses originating from wild birds is greatly needed.

The RNA-dependent influenza A virus RNA polymerase mediates the transcription and replication of the viral genome. Because this enzyme lacks proof-reading capacity, viral replication is characterized by a high mutation rate which allows the evasion of host immune defense and adaptation to new hosts upon interspecies transmission. The adaptation of viral polymerase plays important roles in the virulence and interspecies transmission of influenza viruses, as was shown for H5N1 (Gabriel et al., 2013). In our work, four amino acid substitutions, PB2 E627K, PB2 T23I, PA T97I, and PA S388C, were found in the viral polymerase subunits PB2 and PA. While the PB2 E627K and PA T97I mutations contributed to the enhanced polymerase activity and higher pathogenicity of the P10 virus, and the functions of the other two mutations have yet to be determined. In this study, we found viral polymerase plays important roles in H5N6 virus adaptation to mice.

Receptor binding preference is considered to be a very important factor for influenza A virus transmission in different animal models (Gao et al., 2009; Imai et al., 2012; de Graaf and Fouchier, 2014). The human upper respiratory tract, the primary site of influenza virus replication in humans, was found to be enriched for α2, 6-linked sialic acid receptors, while the human lower respiratory tract was found to be enriched for avian α2, 3-linked sialic acid receptors (Shinya et al., 2006). Binding human α2,6 receptors is a prerequisite for the efficient transmission of influenza virus among humans (Tumpey et al., 2007; Shelton et al., 2011). Because it lacked the ability to bind α2,6 receptors, the mouse-adapted P10 virus was no transmissible in guinea pigs. Herfst et al. (2012) sequentially passaged a genetically modified H5N1 virus that had a preference for α2,6-linked sialic acid receptors, 10 times in ferrets. Among all six airborne-transmissible viruses, in addition to the three introduced mutations (Q222L and G224S in HA and E627K in PB2), two new amino acid substitutions were consistently detected: H103Y and T156A (both in HA) (Herfst et al., 2012). Except for PB2 E627K, these amino acid changes were not found in our study. The Q222L and G224S amino acid mutations not only enhanced the binding of the H5N1 virus to α2,6 receptors, but also increased its affinity for α2,3 receptor, which probably conferred transmissibility to this H5N1 strain. Unlike in our present study, ferrets, thought to most accurately represent human influenza disease, were used as the animal model in this work. The influenza virus receptor distribution in the respiratory tract of ferrets is highly similar to that of humans, as both ferrets and human upper respiratory tracts are predominated by α2,6-linked sialic acids receptors. Hence, ferrets are better suited as an animal model for the selection of amino acid substitutions associated with influenza transmission in humans. Alternatively mice usually used for modeling virulence and pathogenicity, and are thus better suited for the selection of substitutions related to higher virulence and enhanced pathogenicity in virus adaptation to humans.

In summary, we serially passaged an avian H5N6 influenza A virus strain 10 times in mice. P10 showed enhanced pathogenicity, but acquired no transmissibility in mammals. The identified PB2 E627K and PA T97I amino acid substitutions were shown to play important roles in the adaptation of H5N6 to mammals. The HA R239H mutation may be important for the attachment of adapted influenza viruses to their hosts airways; however, the specific effects of these amino acid substitutions on mammalian pathogenicity requires further study. Our study highlights the threat of H5N6, and constant surveillance is needed to monitor the evolution of H5N6 in wild birds and domestic poultry. Specifically, amino acid changes within the HA subunit and the RNA-dependent RNA polymerase complex should be monitored to gain advanced warnings for a better preventing and controlling H5N6 outbreaks.

## Author Contributions

CZ, ZZ, and ZG designed and drafted the work. CZ, ZZ, ZG, JZ, JL, YY, SL, ZW, MZ, YF, XY, LL (Lina Liu), and YZ performed the experiments, analyzed the data, and interpreted the results. JQ, HC, LL (Linna Liu), and YH designed the work and revised it critically.

## Conflict of Interest Statement

The authors declare that the research was conducted in the absence of any commercial or financial relationships that could be construed as a potential conflict of interest.
