# Supplementary material for: Amino Acid Substitutions Associated with Avian H5N6 Influenza A Virus Adaptation to Mice
Source: Front Microbiol. 2017 Sep 15;8:1763. doi: 10.3389/fmicb.2017.01763 (PMC5605651; doi:10.3389/fmicb.2017.01763)
Supplement: Supplementary file 1 [file Table_1.DOC]

Supplementary Materials for

**Amino acid substitutions associated with avian H5N6 influenza A virus adaptation to mice**

Chunmao Zhang^a&^, Zhendong Guo^a&^, Zongzheng Zhao^a&^, Jiajie Zhang^b^, Jiaming Li^a^, Yifei Yang^c^, Shaoxia Lu^b^, Zhongyi Wang^a^, Min Zhi^b^, Yingying Fu^a^, Xiaoyu Yang^b^, Lina Liu^a^, Yi Zhang^a^,Yuping Hua^b*^, Linna Liu^a^*, Hongliang Chai^b^*, Jun Qian^a^*

^a^Military Veterinary Research Institute, Academy of Military Medical Sciences, NO 666 West Liuying Road, Changchun 130122, Jilin, China.

^b^College of Wildlife Resources, Northeast Forestry University, Harbin 150040, China.

^c^Institute of Chinese Materia Medica, China Academy of Chinese Medical Science, Beijing 100700, China

^&^These authors contributed equally to this work.

^*^Authors to whom correspondence should be addressed: qianj1970@126.com(J.Q.), [17758625@163.com(H.C.)](mailto:17758625@163.com(H.C.)), [liulinna7@126.com(L.L.),](mailto:liulinna7@126.com(L.L.),17758625@163.com(H.C.)) yuping_hua@126.com(H.Y.); [Tel./Fax:+86-431-8698-5801(J.Q.)](mailto:qianj1970@126.com(J.Q.);liulinna7@126.com(L.L.)Tel./Fax:+86-431-8698-5801(J.Q.)); +86-431-8698-5856 (L.L.),

Supplementary Table S1

Sequencing primers for influenza virus

| Gene | Primer sequence |
| --- | --- |
| NS-F | ATGGATTCCAATACTGTGTC |
| NS-R | TTAAATAAGCTGAAACGAGAAG |
| M-F | ATGAGTCTTCTAACCGAGG |
| M-R | TTACTCCAATTCTATGTTGAC |
| NA-F | ATGAATCCAAATCAAAAGATAAC |
| NA-R | AGAAACAAGGGTGTTTTTCTTA |
| HA-F | ATGGAGAAAATAGTGCTTCTTC |
| HA-R | TTAAATGCAAATTCTGCATTG |
| NP-F | ATGGCGTCTCAAGGCAC |
| NP-R | TTAATTGTCATACTCCTCTGCAT |
| PB1-F1 | ATGGATGTCAACCCGACTTTA |
| PB1-R1 | ATGTGGTTTTGGTGTATCTTTT |
| PB1-F2 | AGTATTGCCCCTATAATGTTTTC |
| PB1-R2 | CACTAATCACTATTTTTGCCGTC |
| PB2-F1 | AGCAAAAGCAGGTCAAATATA |
| PB2-R1 | TTCTGAAATAGCACCTTGGC |
| PB2-F2 | AAAAGAACAAGTGGGTCGTC |
| PB2-R2 | CAAGGTCGTTTTTAAACAATTC |
| PA-F1 | ATGGAGGACTTTGTGCGAC |
| PA-R1 | GCTCTGAACCCAGCTTGATA |
| PA-F2 | CAGGTGCTGGCAGAACTC |
| PA-R2 | CTATTTCAGTGCATGTGTGAGG |

Fig S1


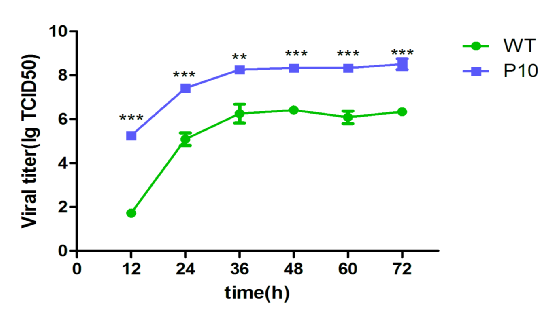


Fig S1 The growth kinetics of P10 and WT H5N6 viruses in MDCK cells. MDCK cells were seeded in the 10cm-culture dishes to 90% confluence the day before infection. The MDCK cells were washed with phosphate-buffered saline (PBS) three times and infected with P10 and WT H5N6 viruses at a multiplicity of infection (MOI) of 0.01 for 1 h at 37 °C, 5% CO_2_ incubator. After incubation, the cells were washed with PBS three times, and 10 ml of opti-MEM reduced serum medium (Gibco, U.S.A.) was added. At 12h, 24h, 36h, 48h, 60h and 72h post incubation, 200 μL of supernatants was taken and stored at -80 °C for titration. Finally, the Viruses in the supernatants collected at different time points were titrated in MDCK cells by tissue culture infective dose (TCID_50_). The experiment was repeated three times independently. Data were analyzed by GraphPad Prism 5.0 software using the two-way ANOVA method, *, P<0.05, **, P<0.01, ***,P<0.001.

Fig S2


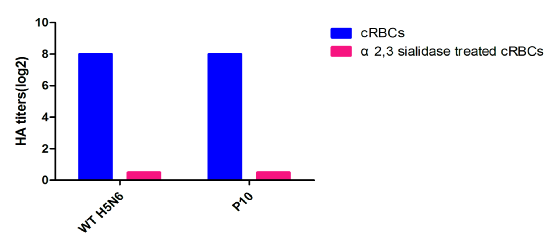


Fig S2 P10 exhibited a preference for α2,3-linked receptors. The receptor binding specificity of P10 and WT H5N6 was determined by HA assay. The cRBCs (chicken red blood cells) were treated with α2,3 sialidase to remove α2,3-linked receptors and α2,6-linked receptors still existed on the surface of treated cRBCs. 0.5% cRBCs and α2,3 sialidase treated cRBCs (tcRBCs) were used to determine the HA titers of influenza viruses P10 and WT H5N6.
